# 20-HETE Participates in Intracerebral Hemorrhage-Induced Acute Injury by Promoting Cell Ferroptosis

**DOI:** 10.3389/fneur.2021.763419

**Published:** 2021-11-12

**Authors:** Ranran Han, Jieru Wan, Xiaoning Han, Honglei Ren, John R. Falck, Sailu Munnuri, Zeng-Jin Yang, Raymond C. Koehler

**Affiliations:** ^1^Department of Anesthesiology and Critical Care Medicine, The Johns Hopkins University, Baltimore, MD, United States; ^2^Department of Biochemistry, University of Texas Southwestern Medical Center, Dallas, TX, United States

**Keywords:** 20-hydroxyeicosatetraenoic acid, intracerebral hemorrhage, ferroptosis, glutathione peroxidase, lipid peroxide

## Abstract

Intracerebral hemorrhage (ICH) is a highly fatal type of stroke that leads to various types of neuronal death. Recently, ferroptosis, a form of cell death resulting from iron-dependent lipid peroxide accumulation, was observed in a mouse ICH model. N-hydroxy-N′-(4-n-butyl-2-methylphenyl)-formamidine (HET0016), which inhibits synthesis of the arachidonic acid metabolite 20-hydroxyeicosatetraenoic acid (20-HETE), has shown a protective effect after ICH. However, the underlying mechanisms of the neuroprotective effect need further investigation. We explored whether 20-HETE participates in ICH-induced ferroptosis *ex vivo* by using hemoglobin-treated organotypic hippocampal slice cultures (OHSCs) and *in vivo* by using a collagenase-induced ICH mouse model. *Ex vivo*, we found that the 20-HETE synthesis inhibitor HET0016 and antagonist 20-6,15-HEDGE reduced hemoglobin-induced cell death, iron deposition, and lipid reactive oxygen species levels in OHSCs. Furthermore, 20-HETE inhibition in OHSCs increased the expression of glutathione peroxidase (GPX) 4, an antioxidant enzyme that serves as a main regulator of ferroptosis. In contrast, exposure of OHSCs to the 20-HETE stable mimetic 20-5,14-HEDGE induced cell death that was significantly inhibited by the ferroptosis inhibitor ferrostatin-1. *In vivo*, HET0016 treatment ameliorated focal deficits, reduced lesion volume, and decreased iron accumulation around the lesion at day 3 and 7 after ICH. In addition, lipid peroxidation was decreased and expression of GPX4 was increased in the HET0016-treated ICH group. The mitogen-activated protein kinase pathway also was inhibited by HET0016 *in vivo*. These results indicate that 20-HETE contributes to ICH-induced acute brain injury in part by activating ferroptosis pathways, thereby providing an upstream target for inhibiting ferroptosis.

## Introduction

Intracerebral hemorrhage (ICH) is a devastating neurologic injury that accounts for 10–15% of all strokes (1). Owing to a lack of treatment options, patients who experience ICH are left with a poor prognosis (2, 3). In the brain, ICH leads to various types of neuronal death, including ferroptosis (4, 5). Ferroptosis is a regulated form of nonapoptotic cell death that results from iron-dependent lipid peroxide accumulation, which can be reduced by glutathione peroxidase 4 (GPX4) activity; specific inhibitors have been designed to block this form of cell death (6, 7). Unrestrained lipid peroxidation, accumulation of redox-active iron, and defective lipid peroxide repair capacity are key hallmarks of ferroptosis (8–10). At the onset of ICH, multiple blood components, including erythrocytes, leukocytes, and platelets, leach into the brain. Lysed erythrocytes release hemoglobin (Hb)/heme, a putative neurotoxin. When it is engulfed by phagocytes, Hb is metabolized into ferrous/ferric iron, which induces the formation of lethal reactive oxygen species (ROS) and lipid peroxidation (11). GPX4, an antioxidant enzyme that reduces peroxidized phospholipids to the corresponding alcohols by using glutathione (GSH) as substrate (12), is one of the central upstream regulators of ferroptosis (13). Loss of GPX4 activity can lead to accumulation of lipid-based ROS, particularly lipid hydroperoxides, and finally cause ferroptosis (14). In addition, activation of the mitogen-activated protein kinase (MAPK) pathway contributes to ferroptotic cell death (15). Emerging data suggest that ferroptosis contributes to ICH-induced secondary brain injury and might be one of most influential cell death types after ICH (4, 5, 16). Therefore, therapy that targets the inhibition of ferroptosis may effectively prevent neuronal death, thereby reducing secondary brain injury after ICH (17).

20-Hydroxy-5,8,11,14-eicosatetraenoic acid (20-HETE) plays an important role in regulating a wide variety of normal physiological functions as well as the pathogenesis of diverse disease conditions (18). This arachidonic acid metabolite synthesized by cytochrome P450 (CYP) 4A/F enzymes is increased in the plasma of patients with ischemic stroke and in cerebrospinal fluid of patients with aneurysmal subarachnoid hemorrhagic stroke (19, 20). In addition, elevated 20-HETE levels are associated with unfavorable outcomes and may be a predictor of neurological deterioration in stroke patients (21, 22). 20-HETE–targeted therapies have been shown to attenuate neuronal death and improve neurological outcomes after ischemic brain injury, independent of vascular effects (23–25). One mechanism by which 20-HETE synthesis within neurons promotes cell death is by stimulating ROS production *via* activation of NADPH oxidase (26). Furthermore, CYP4A expression and 20-HETE were reported to increase in a mouse model of ICH, and its highly selective synthesis inhibitor N-hydroxy-N′-(4-n-butyl-2-methylphenyl)-formamidine (HET0016) (27) was found to be protective after ICH, as assessed by lesion volume and by FluoroJade and TUNEL staining measurements of perilesion neuronal degeneration (28). This protection was associated with decreased activation of microglia and astrocytes and by attenuated formation of protein carbonylation and nitration. However, the effect of 20-HETE synthesis inhibition on lipid peroxidation after ICH was not investigated in this prior study, and the type of cell death induced by 20-HETE in ICH is unknown. Malondialdehyde (MDA) and 4-hydroxy-2-nonenal (4-HNE) are important toxic byproducts of lipid peroxidation, a key characteristic of ferroptosis. *In vivo* treatment with HET0016 can decrease 4-HNE content in vascular tissues in spontaneously hypertensive rats (29) and alleviate the increase in MDA in the perilesional cortex induced by traumatic brain injury (30). However, to our knowledge, no studies have investigated the relationship between 20-HETE and lipid peroxidation-driven ferroptosis in ICH.

Because 20-HETE can stimulate ROS production, we were prompted to investigate whether 20-HETE contributes to ICH-induced neuronal ferroptosis and further explore the mechanism. *Ex vivo* we applied Hb to organotypic hippocampal slice cultures (OHSCs) to mimic the effects of one of the main instigators of ICH injury. *In vivo* we used a collagenase-induced model of ICH in mice. We investigated whether inhibiting 20-HETE reduces biochemical markers of ferroptosis and whether blocking ferroptosis reduces toxic effects of a stable 20-HETE agonist.

## Methods

### Animals and Study Design

Adult male C57BL/6J mice were obtained from the Jackson Laboratory (Bar Harbor, ME USA). The study was conducted in accordance with the ARRIVE and RIGOR guidelines for the use of experimental animals (31). Experimental protocols were approved by the Institutional Animal Care and Use Committee at Johns Hopkins University School of Medicine. Sample size calculations based on our previous (28) and pilot studies indicated that eight mice/group would provide at least 80% power for detecting a 20% decrease in lesion volume at α = 0.05 (two-sided). All efforts were made to ensure minimal pain or discomfort in animals. A total of 235 male C57BL/6J mice were used for *in vivo* experiments and 80 C57BL/6J pups from both sexes were used for OHSC experiments. Among them, 12 mice were excluded because they died before the end of the surgery or shortly thereafter. Animals were randomly assigned to different study groups by using the randomizer form at http://www.randomizer.org. Investigators blinded to the treatment groups evaluated outcomes in all mice and performed analyses.

### ICH Model

Mice at 10–12 weeks of age were anesthetized by 1–3% isoflurane inhalation and ventilated with oxygen-enriched air (20% O_2_:80% air). The right striatum of mice was injected with 0.5 μl of 0.075 U collagenase VII-S (MilliporeSigma, St. Louis, MO, USA) at 0.1 μl per minute. Injections were administered at 0.5 mm anterior and 2.2 mm lateral of the bregma, and 3.0 mm in depth, as previously described (32). Sham-operated mice received the same treatment, including needle insertion, but were not injected with collagenase. Mice that died before the end of the surgery or shortly thereafter were excluded.

### Reagents

HET0016, the 20-HETE stable mimetic N-(20-hydroxyeicosa-5(Z),14(Z)-dienoyl) glycine (20-5,14–HEDGE), and 20-HETE stable antagonist N-(20-hydroxyeicosa-6(Z),15(Z)-dienoyl) glycine (20-6,15–HEDGE) were synthesized in the laboratory of Professor John R. Falck (University of Texas Southwestern Medical Center) (33). Ferrostatin-1, a specific and potent inhibitor of ferroptosis, was obtained from Xcessbio Biosciences (#M60042-2s). Caspase-3 inhibitor VII (#13306) and necrostatin-1 (#11658) were obtained from Cayman Chemical.

### HET0016 Administration

Mice were randomly assigned to each group. HET0016 was administrated intraperitoneally (10 mg/kg per day) beginning at 2 h after surgery. Vehicle-treated mice received the same volume of phosphate-buffered saline containing 10% dimethyl sulfoxide (MilliporeSigma) and 10% Tween-80 (MilliporeSigma). The dose and frequency of HET0016 administration was based on our previous report and pilot studies (28).

### Neurological Deficit Assessment

Behavior tests including the neurological deficit score assessment and wire hanging test were conducted at day 1, 3, and 7 after ICH to assess neurological deficit. A 24-point scoring system that included six tests was applied to assess neurologic deficits of mice. The tests included body symmetry, gait, climbing, circling behavior, front limb symmetry, and compulsory circling (34). Each test was graded from 0 to 4, establishing a maximum deficit score of 24. Mice with a deficit score of more than 20 at 24 h after ICH were excluded from analysis.

For the wire hanging test, a metallic wire (1 mm ×55 cm) was stretched horizontally between two posts, 50 cm above the countertop, which was covered with a pillow to minimize injury caused by falls. The hind limbs of the mice were fixed with adhesive tape to prevent them from using all four paws. The mice were placed on the wire, and latency to fall was recorded. Mice had three trials with a maximum of 180 s per trial, and the longest latency was recorded. Mice that had a latency time <10 s before and after ICH were excluded from analysis.

### Organotypic Hippocampal Slice Cultures

The procedure for culturing OHSCs was similar to that described previously (4, 28). In brief, 7-day-old C57BL/6 pups ware rapidly decapitated. The two brain hemispheres were harvested and cut coronally into 350-μm-thick slices with a Mcllwain tissue chopper. Then, hippocampal slices were immediately separated and placed on a hydrophilic PTFE cell culture insert (PICM0RG50, MilliporeSigma) in a 6-well-plate. Culture medium consisted of 50% DMEM, 25% horse serum, and 25% Hanks' Balanced Salt Solution supplemented with 6.5 mg/mL D-glucose, 25 mM HEPES, 100 U/mL penicillin G potassium, and 100 mg/mL streptomycin sulfate. The slices were cultured in a 37°C incubator with 5% CO_2_. The next day, the medium was changed to 70% DMEM and 5% horse serum. The culture medium was then changed every 2 to 3 days. At 12–14 days *in vitro*, cultured slices were incubated for 24 h in serum-free medium and then exposed to different treatments as designed.

### Propidium Iodide Staining of Slices

After incubation for 24 h in serum-free medium, the cultured slices were exposed to different reagents, including 10 μM Hb (H7379; MilliporeSigma), 10 μM HET0016, 10 μM 20-HETE antagonist (20-6,15-HEDGE), 5–20 μM 20-HETE mimetic (20-5,14-HEDGE), 25 μM caspase-3 inhibitor VII, 10 μM RIP-1–dependent necroptosis inhibitor necrostatin-1, and 10 μM ferrostatin-1 for 18 h. Slices were incubated with 5 μg/ml PI for 30 min before images were taken with an inverted fluorescence microscope (TE2000-E; Nikon, Japan). The PI fluorescence intensity was then measured by ImageJ software (NIH, Bethesda, MD). The fluorescence intensity before Hb or 20-5,14-HEDGE treatment was recorded as P0h, and that after the treatment was P18h. Maximum PI (Pmax) staining was taken as that induced by 100 μM N-methyl-D-aspartate. Cell death was calculated by the formula: (P18h–P0h)/(Pmax–P0h) × 100%.

### Lipid Peroxidation Detection

The Image-iT lipid peroxidation kit (C10445; Life Technologies, Carlsbad, CA, USA) based on BODIPY 581/591 C11 reagent was used as an indicator of OHSC lipid peroxidation. After incubation with Hb and HET0016 or 20-6,15-HEDGE for 6 h, all treated slices were incubated with Image-iT Lipid Peroxidation Sensor at a final concentration of 10 μM for 30 min at 37°C. Some slices were observed by microscopy and others were lysed and emission fluorescence intensity detected at 590 and 510 nm on a microplate reader.

A lipid peroxidation colorimetric/fluorometric assay kit (K739; BioVision, Milpitas, CA, USA) was used to detect MDA levels in cell lysates of brain (4 mm coronal sections from the major hemorrhagic territory) 3 days after ICH according to the manufacturer's instructions. Briefly, MDA in the sample reacted with thiobarbituric acid (TBA) to generate an MDA-TBA adduct. The MDA-TBA adduct was collected and quantified colorimetrically with a microplate reader (OD 532 nm). The amount of MDA was calculated according to the standard curve and expressed as nmol/mg protein.

4-HNE is another byproduct of lipid peroxidation. For its measurement, we used western blot analysis and an ELISA kit (STA-838; Cell Biolabs, San Diego, CA, USA). The ELISA was carried out according to the manufacturer's instructions at day 3 after ICH. In brief, after coating an HNE conjugate on an ELISA plate, samples lysed from a 4 mm coronal brain section or HNE-BSA standards were added for incubation. Then an anti-HNE polyclonal antibody was added, followed by a horseradish peroxidase-conjugated secondary antibody. The quantity of HNE adduct in protein samples was determined by comparing its absorbance at 450 nm with that of a known HNE-BSA standard curve. The results were expressed as percentage relative to sham group.

GSH from brain tissue was measured with a GSH assay kit (DIGT-250; BioAssay Systems, Hayward, CA, USA) with the improved 5,5′-dithiobis(2-nitrobenzoic acid) (DTNB) method at day 3 after ICH. The brain tissues were lysed in cold buffer containing 50 mM MES and 1 mM EDTA and the supernatant collected for assay. After deproteination, the samples reacted with DTNB to form a yellow product. The concentration of GSH was determined by comparing its optical density, measured at 412 nm, with that of calibrator and blank. The results were expressed as percentage relative to sham group.

### Lesion Volume Analysis

At day 1, 3, or 7 after ICH, mice were euthanized by deep anesthesia with isoflurane and perfused transcardially before brains were collected. Each brain was post-fixed in 4% paraformaldehyde overnight and then transferred to 30% sucrose. Then, 25 μm-thick coronal brain sections spaced 150 μm apart were cut with a cryostat microtome (Leica, Wetzlar, Germany). Sections were mounted on slides and left to dry overnight at room temperature. Lastly, the slides were stained with cresyl violet (for neurons, MilliporeSigma) and luxol fast blue (for myelin). Coronal images were digitized with a microscope (Nikon Eclipse 90i) and analyzed with ImageJ software. The lesion volume was calculated as the sum of the damaged area multiplied by the distance between the sections (150 μm). The total lesion volume was also corrected to account for brain edema: corrected lesion volume = volume of non-hemorrhagic hemisphere–(volume of hemorrhagic hemisphere–lesion volume).

### Iron Staining

Iron staining was performed with OHSCs18 h after exposure to Hb with or without HET0016 and at day 3 and 7 after ICH. We applied 3,3′-diaminobenzidine (DAB)-enhanced Perls' staining (HT20; MilliporeSigma) to detect iron deposition according to the manufacturer's instructions with minor modification. OHSCs and brain slices were fixed and washed before being incubated with working solution, which was prepared by mixing equal volumes of potassium ferrocyanide solution and hydrochloric acid solution. Then the samples were blocked in 0.3% hydrogen peroxide and incubated with DAB (SK-4100; Vector Laboratories, Burlingame, CA, USA) for 3 min. Next, the nuclei were stained in working pararosaniline solution. After the slices were dehydrated and mounted on slides, images were taken under a microscope. Images were captured from five optical fields (20 × 10 magnification) in each of three sections per animal. Iron-positive cells were counted with ImageJ software.

### 20-HETE Detection

After incubation for 24 h in serum-free medium, the cultured slices were exposed to Hb with or without HET0016 for 18 h. Then, 20-HETE was detected by using a 20-HETE ELISA kit (20H1; Detroit R&D, Detroit, MI, USA). To prepare OHSCs for the detection, we added a solution containing a final concentration of 0.1 mM triphenylphosphine onto each insert, scraped the OHSCs, and transferred the sample into 1.5 ml tubes. After saponification, the whole homogenate was acidified with acetic acid to a pH of ~3–4. Then the samples were extracted with an equal amount of ethyl acetate three times to pool the organic phase. After the organic phase was dried with nitrogen gas, the remaining residue was dissolved in N,N-dimethyl-formamide. Finally, this solution was diluted with sample dilution buffer, and 20-HETE was detected by ELISA as indicated by the manufacturer. The results were determined by comparing its absorbance at 450 nm with that of a known 20-HETE standard curve.

### Western Blot

We extracted total protein from OHSCs 18 h after applying different treatments and from 4 mm coronal sections of the major hemorrhagic territory at day 1 and day 3 after ICH by using T-PER tissue protein extraction reagent (78510; Thermo Fisher Scientific, Waltham, MA) supplemented with protease inhibitor cocktail and phenylmethylsulfonyl fluoride. Total protein was quantified by bicinchoninic acid assay (23227; Thermo Scientific), and equal amounts were separated by sodium dodecyl sulfate-polyacrylamide gel electrophoresis before transfer onto polyvinylidene fluoride membranes. Membranes were incubated with the following antibodies overnight: GPX4 (1:1,000, ab125066; Abcam, Cambridge, UK), HNE (1:1000, 393207; MilliporeSigma), cyclooxygenase (COX)-2 (1:500, 35-8200; Invitrogen, Carlsbad, CA, USA), SAPK/c-Jun NH2-terminal kinase (JNK; 1:1000, 9252; Cell Signaling Technology, Danvers, MA, USA), phospho-SAPK/JNK (T183/Y185; 1:1000, 4668; Cell Signaling Technology), p44/42 MAPK extracellular signal- regulated kinase (ERK)1/2 (1:1000, 4695; Cell Signaling Technology), phospho-p44/42 MAPK_ERK1/2 (T202/Y204; 1:1000, 4370; Cell Signaling Technology), p38 MAPK (1:1000, 8690; Cell Signaling Technology), phospho-p38 MAPK (T180/Y182; 1:1000, 4511; Cell Signaling Technology), vinculin (1:1000, 4650; Cell Signaling Technology), β-actin (1:2000, sc-47778; Santa Cruz Biotechnology, Dallas, TX). Then appropriate horseradish peroxidase-conjugated secondary antibodies were applied to detect bands under an ImageQuant ECL imager (GE Healthcare, Chicago, IL, USA) or iBright™ CL1000 imaging system (Themo Fisher Scientific). No blot was stripped and re-used for antibody probing in this study. Bands were analyzed by ImageJ software. All data were normalized to the corresponding loading control and expressed as fold change to the sham group in mice or control group in OHSCs.

### Immunofluorescence

PI-stained OHSCs were fixed in 4% paraformaldehyde in 6-well-plates for 2 h. Then the slices were permeabilized by 0.3% triton for 30 min and blocked with 10% goat serum for 2 h at room temperature. Next, different antibodies were added to each insert, including anti-neuronal nuclei (NeuN) antibody (1:500, 12943S; Cell Signaling Technology), anti-glial fibrillary acidic protein (GFAP; 1:500, 13-0300; Life Technologies), and anti-ionized calcium binding adaptor molecule 1 (Iba1) antibody (1:500, 019-19741; Wako, Osaka, Japan), and incubated overnight at 4°C. After appropriate secondary antibody incubation, the nuclei were labeled by DAPI (H-1200; Vector Laboratories). Pictures were taken with an inverted microscope (Nikon Eclipse 90i). We performed immunofluorescence to assess GPX4 level at day 3 after ICH and the cultured slices were exposed to Hb with or without HET0016 for 18 h before GPX4 staining. The procedure to stain GPX4 (1:200, ab125066; Abcam) in OHSCs and brain sections was similar to that described above but without PI staining. The specificity of the primary antibodies in immunostaining was assessed by performing the experiments without the primary antibodies.

### Statistical Analysis

Data are presented as mean ± SD or median ± confidence interval. The Mann–Whitney *U*-test was applied to compare ranked data from neurological scores or nonparametric data between two groups. For other normally distributed data from two-group comparisons, we applied a two-tailed Student's *t*-test. Differences among multiple groups were analyzed by one-way ANOVA followed by Bonferroni or Dunn's *post-hoc* test. All analyses were carried out with GraphPad Prism 7 software. *p* < 0.05 was considered statistically significant.

## Results

### 20-HETE Contributes to Hb-Induced Cell Death in OHSCs

**Figure 1:**
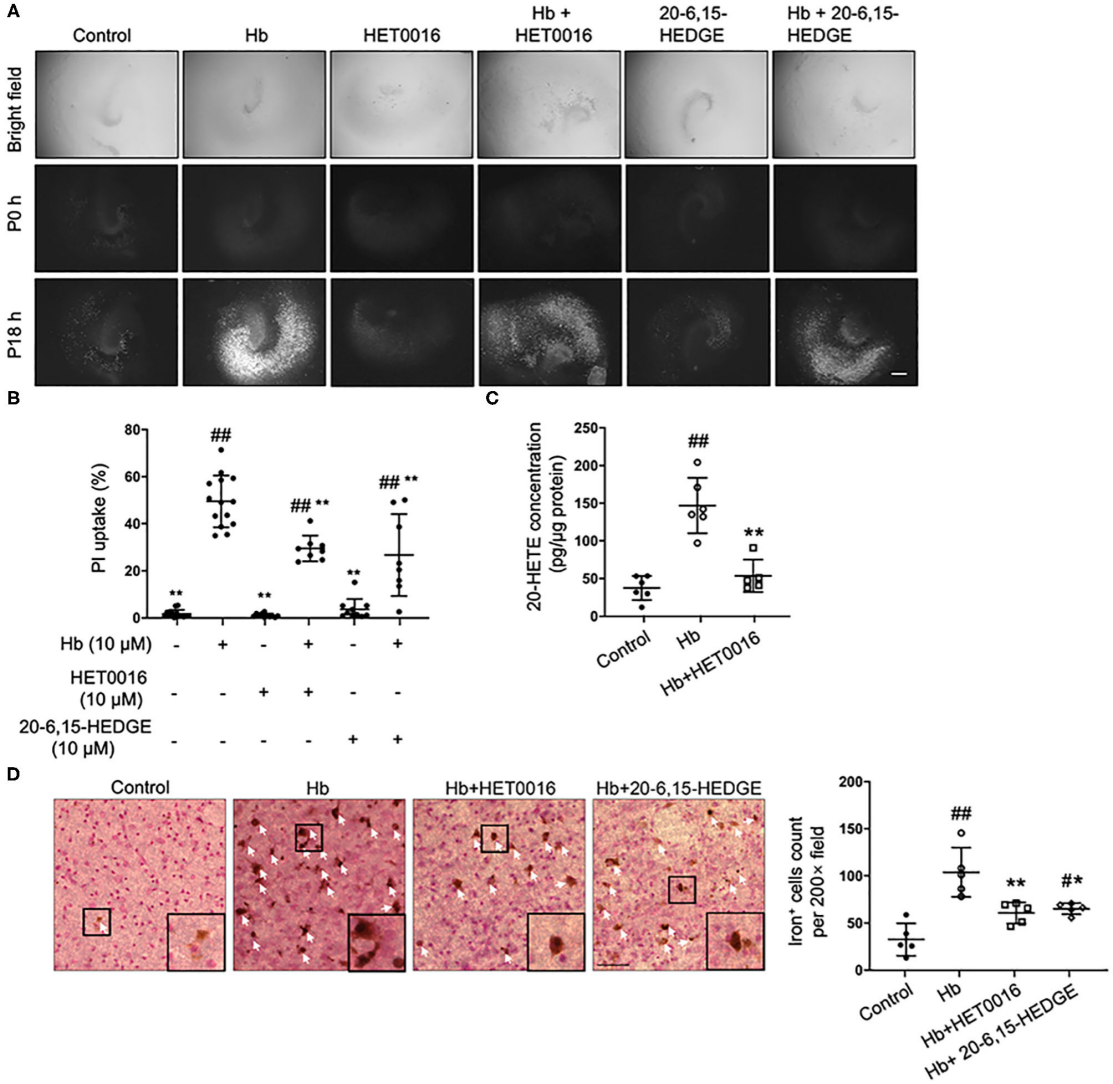
20-HETE participates in hemoglobin (Hb)-induced cell death in organotypic hippocampal slice cultures (OHSCs). **(A)** OHSCs were exposed to 10 μM Hb. HET0016 (10 μM), a 20-HETE synthesis inhibitor, or 20-6,15-HEDGE (10 μM), a 20-HETE antagonist, was added along with Hb for 18 h. OHSCs treated with HET0016 or 20-6,15-HEDGE alone and a vehicle-treated group served as controls. Propidium iodide (PI) staining was performed before (P0h) and 18 h after (P18h) reagent treatment. Scale bar is 500 μm. **(B)** Quantification of **(A)**. Cell death was evaluated as PI uptake percentage. Hb-induced cell death was significantly lowered by both HET0016 and 20-6,15-HEDGE. *n* = 8–14 slices/group. **(C)** Hb significantly increased 20-HETE content in OHSC lysates at 18 h, but HET0016 prevented this increase. *n* = 5–6 from more than 25 slices per group. **(D)** OHSCs were exposed to Hb with or without HET0016 or 20-6,15-HEDGE for 18 h, after which iron deposition was assessed. Representative images are shown along with examples of iron-positive cells at high magnification (insets). Arrows indicate iron-positive cells. Scale bar is 50 μm. Both HET0016 and 20-6,15-HEDGE significantly reduced Hb-induced iron accumulation. *n* = 5 slices/group. ^#^*P* < 0.05, ^##^*P* < 0.01 vs. Control group; *P < 0.05, **P < 0.01 vs. Hb group. One-way ANOVA followed by appropriate *post hoc* test was applied. Data are expressed as mean ± SD.

As a pre-animal experimental phase for physiologic and pathologic brain research, OHSCs offer outcomes that are relatively closer to those of whole-animal studies than outcomes obtained from cell culture *in vitro* (35). In addition, OHSCs have been successfully used to investigate the pathophysiology of intracerebral hemorrhage (4). Therefore, we chose Hb-treated OHSCs as an *ex vivo* ICH model. First, HET0016, a 20-HETE–specific synthesis inhibitor, and 20-6,15-HEDGE, a stable antagonist of 20-HETE were applied. As shown in Figures 1A,B, both 20-HETE inhibitors significantly reduced Hb-induced cell death in OHSCs, in agreement with previous work (28). We extended the previous work by showing that Hb can induce 20-HETE production in OHSCs, whereas HET0016 application significantly reduced it (Figure 1C). Furthermore, exposure to Hb induced more than double amounts of iron deposition relative to that in control slices, but treatment with either 20-HETE inhibitor lessened the iron deposition (Figure 1D).

### 20-HETE Promotes Ferroptosis in *ex vivo* ICH Model

**Figure 2:**
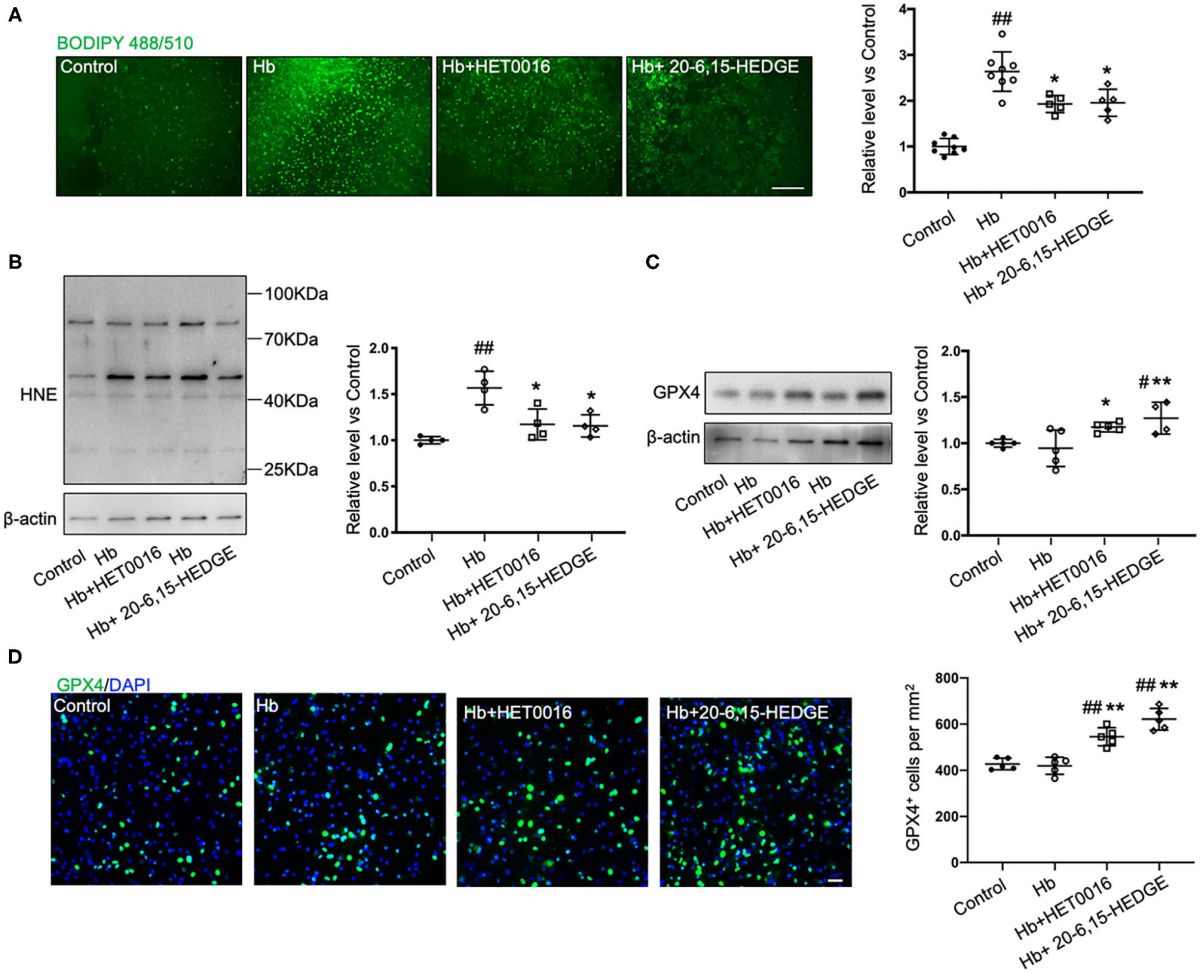
20-HETE promotes ferroptosis in *ex vivo* ICH model. Organotypic hippocampal slice cultures (OHSCs) were exposed to 10 μM hemoglobin (Hb) with or without 10 μM HET0016 or 10 μM 20-6,15-HEDGE. **(A)** Lipid peroxidation level was detected with BODIPY 581/591 C11 reagent 6 h after incubation with different reagents. Representative fluorescent images and fluorescent intensity quantification relative to control slices showed an increased lipid peroxidation state with Hb exposure that was attenuated by HET0016 or 20-6,15-HEDGE co-treatment. Scale bar is 200 μm. *n* = 5–8 slices/group. **(B,C)** Western blotting of OHSC total protein extract was carried out to assess the level of 4-hydroxy-2-nonenal (4-HNE) adducted protein **(B)** and glutathione peroxidase (GPX) 4 **(C)** 18 h after incubation with different reagents. The expression was normalized relative to the control group. 20-HETE inhibition decreased 4-HNE adducted protein level and increased GPX4 expression. *n* = 4–5 from at least 20 slices/group. **(D)** Immunofluorescence result of GPX4 expression at 18 h after exposure to different reagents in fixed slices. Bar is 30 μm. *n* = 5 slices/group. ^#^*P* < 0.05, ^##^*P* < 0.01 vs. Control group; *P < 0.05, **P < 0.01 vs. Hb group. One-way ANOVA followed by appropriate *post hoc* test was applied. Data are expressed as mean ± SD.

To further explore how 20-HETE inhibition affects ferroptosis, we measured lipid peroxidation markers in slices exposed to Hb with or without 20-HETE inhibitors. We detected the changes in lipid peroxidation with the C11 BODIPY reagent measured at 6 h and by assessing 4-HNE levels at 18 h. Both 20-6,15-HEDGE and HET0016 attenuated Hb-induced lipid peroxidation markers (Figures 2A,B), indicating that 20-HETE participates in Hb-induced lipid peroxidation in slice cultures.

Next, we examined the effects of 20-HETE inhibition on the protein expression of GPX4, a central regulator that serves to inhibit ferroptosis. Western blotting showed that in OHSCs exposed to Hb for 18 h, both 20-HETE inhibitors significantly increased GPX4 expression (Figure 2C). Furthermore, images of GPX4 immunostaining in OHSCs confirmed this result (Figure 2D). These findings are consistent with the hypothesis that inhibition of 20-HETE decreases Hb-induced ferroptosis in brain slice cultures.

### 20-HETE–Induced Cell Death Is Mitigated by Inhibiting Ferroptosis

**Figure 3:**
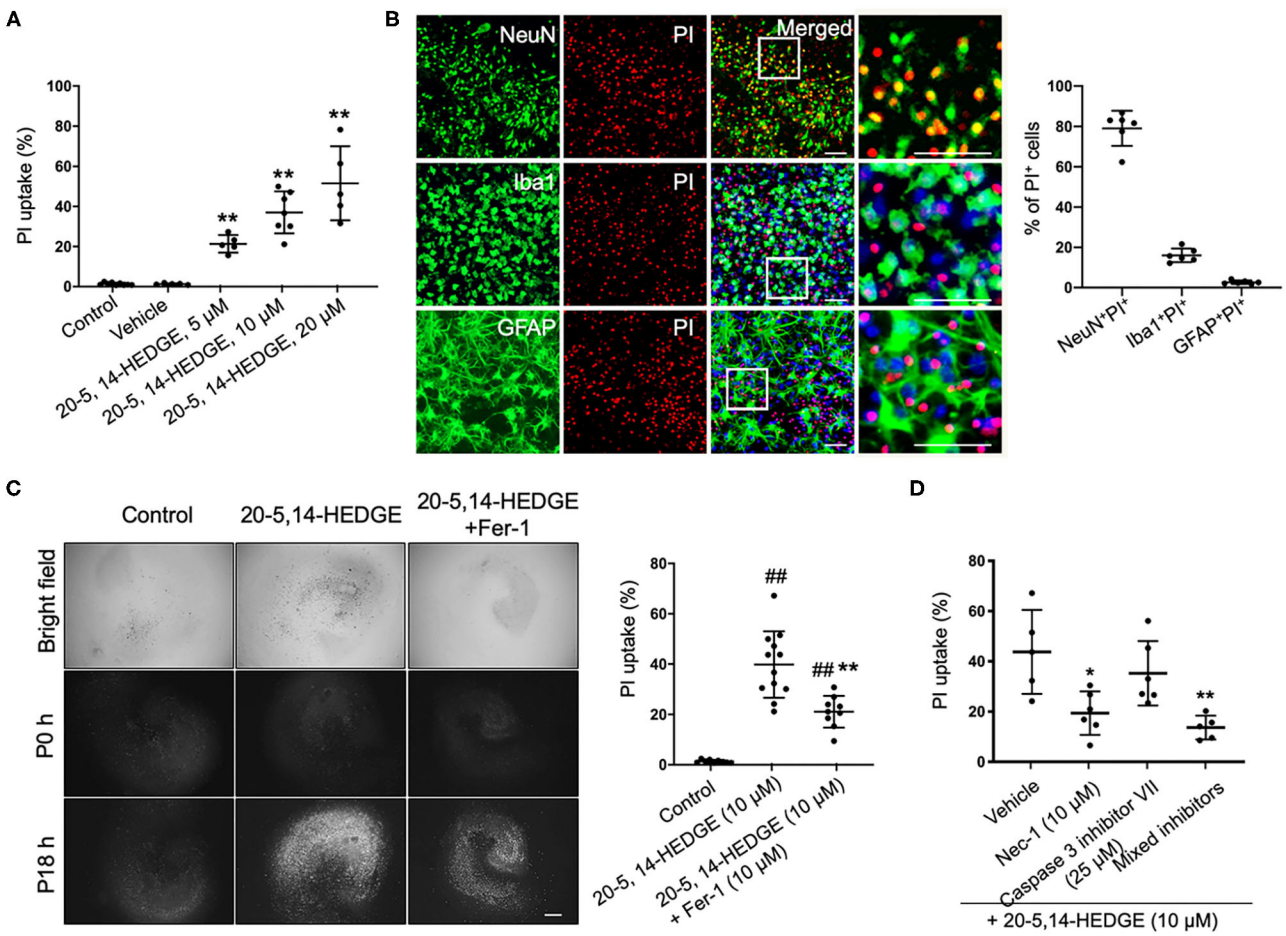
20-HETE–induced cell death is mitigated by inhibiting ferroptosis. **(A)** The stable 20-HETE mimetic 20-5,14-HEDGE at indicated concentrations or vehicle was added to organotypic hippocampal slice cultures (OHSCs), and cell death was assessed by PI staining 18 h later. All three doses of 20-5,14-HEDGE induced significant cell death. The dose of 10 μM was chosen for subsequent experiments because it produced cell death similar to that produced by Hb. *n* = 5–9 slices/group. **(B)** Fixed slices were immunostained with NeuN, Iba1, and GFAP (green) to assess the distribution of neurons, microglia, and astrocytes among PI-labeled dead cells. The percentage of PI^+^ neurons (PI^+^NeuN^+^) was more than PI^+^ microglia (PI^+^Iba1^+^) and PI^+^ astrocytes (PI^+^GFAP^+^). Bar is 50 μm. *n* = 6 slices/group. **(C)** The effect of ferrostatin-1 (Fer-1; 10 μM) on 20-5,14-HEDGE-induced cell death was measured by PI staining. Fer-1 significantly alleviated 20-HETE mimetic-induced cell death. Bar is 500 μm. *n* = 9–12 slices/group. **(D)** Effect of cell death inhibitors caspase 3 inhibitor VII (apoptosis inhibitor, 25 μM), necrostatin-1 (Nec-1, necroptosis inhibitor, 10 μM), and a combination of Fer-1, caspase 3 inhibitor, and Nec-1 on 20-5,14-HEDGE–induced cell death was assessed by PI staining. *n* = 5–6 slices/group. *P < 0.05, **P < 0.01 vs. 20-5,14-HEDGE Vehicle group; ^##^*P* < 0.01 vs. Control group. One-way ANOVA followed by appropriate *post-hoc* test was applied. Data are expressed as mean ± SD.

To more directly observe whether 20-HETE can induce ferroptosis, we applied the stable 20-HETE mimetic 20-5,14-HEDGE to OHSCs and assessed cell death. PI staining showed that 20-5,14-HEDGE significantly and dose-dependently induced cell death (Figure 3A). Moreover, the dead cells consisted mainly of neurons, as co-staining of PI and cell markers indicated predominant colocalization with NeuN (Figure 3B). Figure 3C shows that 20-HETE-induced cell death was significantly ameliorated by the ferroptosis-specific inhibitor ferrostatin-1. To determine how the magnitude of protection with ferrostatin-1 compared to other cell death pathway inhibitors, we treated 20-5,14-HEDGE–exposed OHSCs with the caspase-3 inhibitor VII and the RIP-1–dependent necroptosis inhibitor necrostatin-1. Necrostatin-1 had a protective effect comparable to that of ferrostatin-1, whereas the caspase-3 inhibitor exerted a small, non-significant effect (Figure 3D). The combination of all three inhibitors had the greatest protective effect. These data indicate that ferroptosis is one of the major contributors to 20-HETE–induced cell death, although cross-talk between cell death signaling pathways likely exists.

### HET0016 Ameliorates ICH Severity

**Figure 4:**
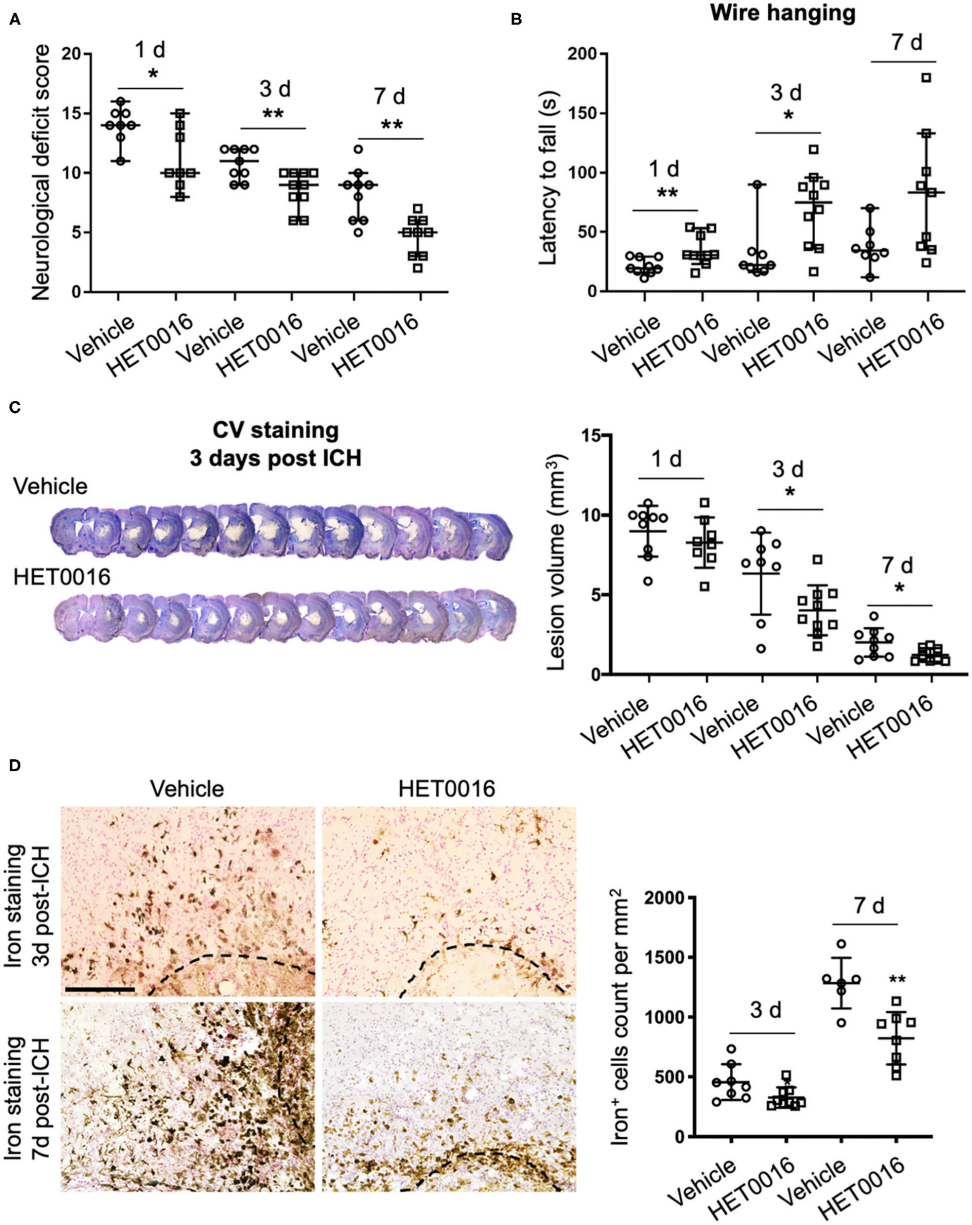
HET0016 ameliorates ICH injury severity in mice. **(A)** HET0016 treatment improved neurological deficit scores of mice at 1, 3, and 7 days after ICH. *n* = 8–10/group. **(B)** HET0016-treated ICH mice exhibited longer latency to fall in the wire-hanging test than did vehicle-treated mice at 1, 3, and 7 days after ICH. *n* = 8–10/group. **(C)** Examples of cresyl violet (CV)/luxol fast blue–stained serial brain sections from vehicle- and HET0016-treated mice 3 days after ICH. Lesion volume was similar on day 1 but significantly less on days 3 and 7 in the HET0016-treated group. *n* = 8–10/group. **(D)** Representative images of 3,3′-diaminobenzidine (DAB)-enhanced Perls' staining shows iron deposition at 3 and 7 days after ICH. Scale bar is 200 μm. The number of iron-positive cells was lower in the HET0016-treated group than in the vehicle-treated group. *n* = 6–9/group. *P < 0.05, **P < 0.01. Mann–Whitney *U*-test **(A,B)** or unpaired *t*-test **(C,D)** was applied between vehicle and HET0016 groups at each time point. Data are expressed as median with confidence interval **(A,B)** or mean ± SD **(C,D)**.

To corroborate our *in vitro* findings *in vivo*, we investigated whether treatment with HET0016 reverses ICH-induced ferroptosis in a collagenase-induced ICH model. Previous work demonstrated that the brain concentration of 20-HETE increased after ICH and that administration of HET0016 blocked this increase and reduced the lesion size at 3 days (28). Here, we first confirmed that HET0016 protected mice with ICH, but we prolonged the time course to 7 days after surgery. Mice treated with HET0016 exhibited improved neurologic deficit scores (Figure 4A), prolonged latency to fall in the wire-hanging test (Figure 4B), and smaller lesion volumes (Figure 4C) after ICH relative to those in the vehicle-treated group. These collective data indicate that HET0016 protected the brain for at least 7 days post-ICH. In addition, our team previously reported by that HET0016 administration can alleviate early neurodegeneration, as assessed by Fluoro-Jade B staining and the TUNEL (terminal deoxynucleotidyl transferase dUTP nick end labeling) method at 3 days after ICH (28). Here, because ferroptosis is driven by the amount of mobile iron, we measured the effect of HET0016 on ICH-induced iron overload in brain sections by DAB-enhanced Perls' staining. The number of iron-positive cells was counted per 200 × field and compared. As shown in Figure 4D, treatment with HET0016 significantly reduced the iron overload at 3 and 7 days post-ICH.

### HET0016 Decreases ICH-Induced Lipid Peroxidation

**Figure 5:**
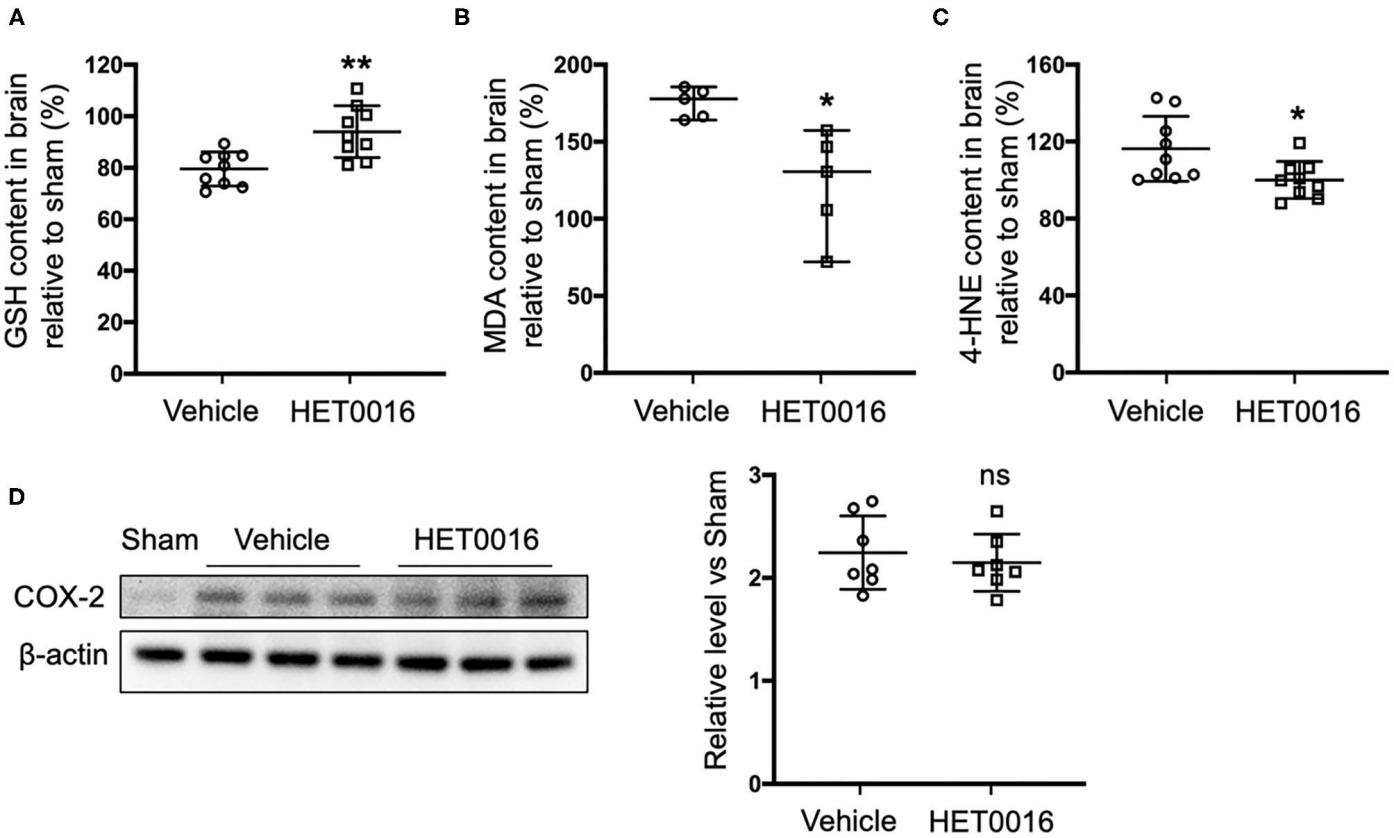
HET0016 decreases lipid peroxidation level after ICH mice. Glutathione (GSH) **(A)**, malondialdehyde (MDA) **(B)**, and 4-hydroxy-2-nonenal (4-HNE) **(C)** content in brain 3 days after ICH. HET0016 reversed the ICH-induced reduction in GSH and increases in MDA and 4-HNE. *n* = 9 **(A,C)** or 5 **(B)** per group. **(D)** Brain tissue surrounding the hematoma was harvested 3 days after ICH, and total protein was extracted for western blotting. After ICH, cyclooxygenase-2 (COX-2) levels increased to a similar extent in the vehicle and HET0016 groups relative to that in the Sham group. *n* = 7/group. *P < 0.05, **P < 0.01 vs. Vehicle group. Mann–Whitney *U*-test **(B)** or unpaired *t*-test **(A,C,D)** was applied. Data are expressed as median with confidence interval **(B)** or mean ± SD **(A,C,D)**.

Next we investigated the effect of HET0016 on the accumulation of lipid peroxides 3 days after ICH. Treatment with HET0016 significantly blunted ICH-induced increases in the levels of the lipid peroxidation markers MDA and 4-HNE, and attenuated the ICH-induced decreases in GSH, which acts as a cofactor of GPX4 to inhibit ferroptosis (Figures 5A–C). However, the expression of COX-2, another enzyme that uses arachidonic acid as a substrate and can metabolize 20-HETE, increased after ICH, but this increase was not affected by HET0016 treatment (Figure 5D).

### HET0016 Affects Ferroptosis Pathway After ICH

**Figure 6:**
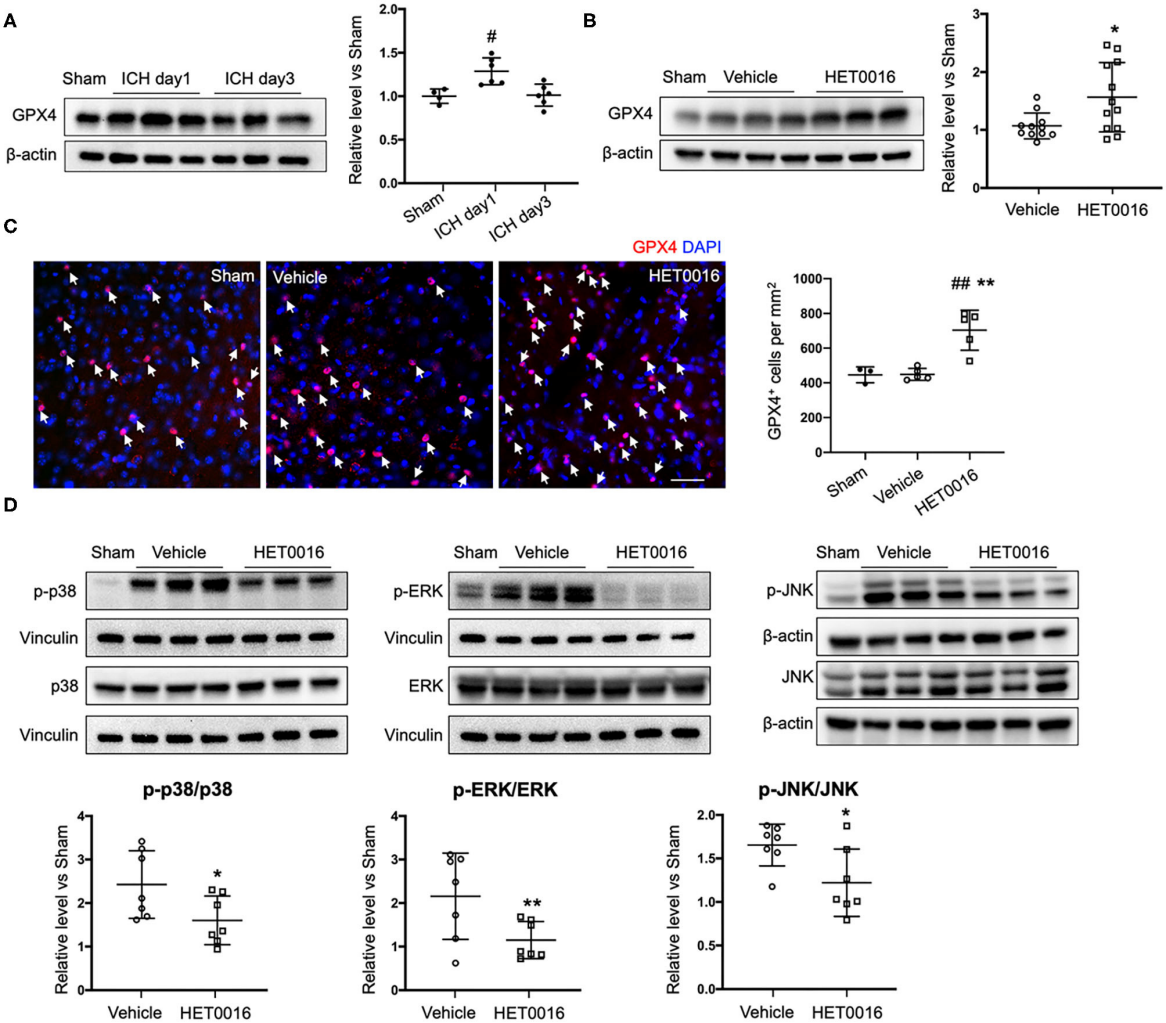
HET0016 affects ferroptosis pathway after ICH in mice. **(A)** Brain tissue surrounding the hematoma was harvested 1 and 3 days after ICH, and total protein was extracted for western blotting. Glutathione peroxidase (GPX) 4 expression was significantly higher at day 1 post-ICH than it was in the sham group and then declined to baseline at day 3 post-ICH. *n* = 4 in sham group, *n* = 6 in ICH groups. **(B)** Western blot analysis shows GPX4 expression in brain tissues from different groups at day 3 after ICH. GPX4 level was significantly higher in the HET0016-treated group than in the vehicle-treated group. *n* = 12/group. **(C)** Immunofluorescence staining was carried out 3 days after ICH for GPX4 (red) and nuclei (blue). Arrows indicate the GPX4–positive cells. Scale bar is 30 μm. Quantification of GPX4 positive cell density is shown. **(D)** Total and phospho-P38/ERK1/2/JNK expression 3 days after ICH. Values were calculated as ratios of phospho- to total-p38/ERK1/2/JNK and further normalized to vinculin or β-actin loading controls. The ratios of p-p38/p38, p-ERK1/2/ERK1/2, and p-JNK/JNK were significantly lower in the HET0016-treated group than in the vehicle-treated group. *n* = 7/group. ^#^*P* < 0.05, ^##^*P* < 0.01 vs. Sham group; *P < 0.05, **P < 0.01 vs. Vehicle group. One-way ANOVA followed by appropriate *post-hoc* test was applied for multiple comparison and *t*-test for two group comparison. Data are expressed as mean ± SD.

We also investigated changes in the ferroptosis signaling pathway. GPX4 protein was significantly increased in coronal sections at 1 day after ICH, compared to that in sham mice, but returned to baseline levels at 3 days (Figure 6A). However, with HET0016 treatment, GPX4 levels remained elevated at 3 days of recovery (Figure 6B). This elevation was confirmed by immunofluorescence staining (Figure 6C). These *in vivo* data are consistent with our finding that HET0016 inhibited Hb-induced ferroptosis in OHSCs. Furthermore, previous research has shown that MAPK signaling is associated with ferroptosis induced by erastin (36, 37) and by oxygen-glucose deprivation and reoxygenation (38). In addition, 20-HETE was reported to affect the MAPK pathway (25, 39), which mainly includes ERK, p38 MAPK, and c-Jun NH2-terminal kinase (JNK). We explored components of the MAPK pathway 3 days after ICH and found enhanced ERK1/2, p38 MAPK, and JNK phosphorylation in the vehicle-treated ICH group. These increases were attenuated significantly after HET0016 treatment (Figure 6D).

## Discussion

In this study, we examined whether 20-HETE participates in ICH-induced cell ferroptosis. We found that (1) Hb-induced ferroptosis in OHSCs can be ameliorated by 20-HETE inhibitors; (2) 20-HETE induces ferroptosis in OHSCs; and (3) Inhibition of 20-HETE synthesis improves ICH outcome and attenuates markers of ferroptosis, such as mobile iron, lipid peroxidation, and decreased GPX4. Furthermore, MAPK pathway activation is reduced in ICH mice treated with a 20-HETE inhibitor.

We selected ferroptosis and 20-HETE as breakthrough points to investigate the mechanism underlying the neurotoxicity of ICH from a new perspective. First, we verified that 20-HETE inhibition can reduce ICH-induced cell death and decrease iron accumulation *in vitro* and *in vivo* (Figures 1, 4). Iron accumulation within the perihematoma area contributes to secondary injury after ICH (40–42) by inducing the formation of lethal ROS and lipid peroxidation (11, 43). Therefore, we further explored whether 20-HETE inhibition reduces lipid peroxidation after ICH. *In vitro*, both the 20-HETE synthesis inhibitor and the 20-HETE antagonist suppressed lipid peroxidation markers in OHSCs exposed to Hb (Figure 2). Likewise, HET0016 treatment *in vivo* inhibited lipid peroxidation markers such as MDA and 4-HNE 3 days after ICH (Figure 5). In patients, MDA is significantly elevated in serum within 4 h of ICH diagnosis and is highly associated with 30-day mortality (44). Taken together, our findings indicate that 20-HETE inhibition improves ICH outcome and decreases lipid peroxidation after ICH both *in vitro* and *in vivo*. Elevated COX-2 level also can be associated with lipid peroxidation and ferroptosis (4). However, we found no difference in COX-2 expression between vehicle- and HET0016-treated groups (Figure 5D). As 20-HETE is a known substrate for COX-2 (45, 46), and COX-2 inhibition can increase 20-HETE levels (47), we hypothesized that reduction in 20-HETE might promote COX-2 expression in a negative feedback fashion. Whether a combination of 20-HETE and COX-2 inhibition would achieve a stronger protective effect after ICH needs further investigation.

Next, to further assess the relationship between 20-HETE and ferroptosis, we incubated OHSCs with a stable 20-HETE mimetic at a concentration sufficient to induce ROS production and neuronal cell death (26). Notably, ferrostatin-1, a specific and potent inhibitor of ferroptosis, significantly blocked this neuronal cell death (Figure 3C). This result is consistent with the hypothesis that 20-HETE induction after ICH can lead to ferroptosis. It has been established that various forms of cell death are not independent but rather intricately interconnected, forming a signaling network that can produce a spectrum of cell death modalities and damage after ICH (48). Therefore, we applied a combination of cell death inhibitors to 20-HETE mimetic-treated OHSCs. Interestingly, the RIP1-dependent necroptosis inhibitor was also effective, and a caspase-3 inhibitor was marginally effective at reducing the consequent cell death (Figure 3D). These findings indicate that 20-HETE can induce different types of cell death and that the reduction in Hb-induced cell death produced by the 20-HETE synthesis inhibitor and antagonist could be attributable not only to reducing ferroptosis but also to reducing cell death through other signaling pathways.

GPX4 is a central regulator that inhibits ferroptosis (13). In the brains of rats subjected to autologous whole blood-induced ICH, GPX4 level was significantly reduced from 12 to 48 h. The level reached a nadir at 24 h but by day 3 was indistinguishable from that of shams (49). In contrast, Alim et al. (50) reported that GPX4 protein was significantly elevated in brain tissue from mice 24 h after collagenase-induced ICH. Our results with the same mouse model confirm the latter findings (Figure 6A). It is possible that the decreased GPX4 in the blood-induced ICH model led to excessive ROS and oxidative lipid damage that finally induced ferroptosis (49), whereas the elevation of GPX4 after collagenase-induced ICH was part of an adaptive response to protect neurons from ferroptotic stimuli (50). Although it is unclear why these two ICH models in two animal species had opposing effects on GPX4 expression at 24 h, we found that GPX4 level had returned to the control level at day 3 after ICH, a time when GPX4 also had returned to baseline in the rat whole blood ICH model. Nevertheless, at this time point, HET0016 treatment produced an increase in GPX4 expression, which we interpret as an effect of HET0016's ability to limit ferroptosis. The increase in GSH and decrease in lipid peroxidation (Figure 5) are consistent with HET0016 limiting ferroptosis. Furthermore, in contrast to a previous report that GPX4 was elevated in primary cortical neurons treated with hemin for 8 h (50), we detected no increase in GPX4 in OHSCs exposed to Hb for 18 h (Figure 2C). The treatment time, in addition to the use of different reagents and cell systems, may be a critical factor leading to these different outcomes. However, HET0016 upregulated GPX4 level in the Hb-treated OHSC, reflecting its inhibition of ferroptosis in association with the reduced cell death. Collectively, these results indicate that inhibition of 20-HETE synthesis can suppress the ferroptosis pathway in both *in vivo* and *in vitro* ICH models.

The mammalian family of MAPKs includes mainly ERK (including ERK1/2), p38 MAPK, and JNK (including JNK1, JNK2, and JNK3). The ERK-dependent signaling pathway is required for ferroptosis in solid cancer cells (15, 51), and enhanced phopho-ERK1/2, a biochemical feature consistent with ferroptosis, was observed in neurons *in vitro* after hemin exposure and *in vivo* after ICH (5). We also observed an increase in the level of phopho-ERK1/2 at day 3 after ICH (Figure 6D). HET0016 administration decreased the phosphorylation of ERK1/2, consistent with its ability to inhibit ferroptosis. Similarly, HET0016 was able to decrease phopho-ERK1/2 in a hypoxia-ischemia model of neuronal injury in association with a protective effect (25). Recently, high mobility group box 1 was demonstrated to regulate ferroptosis through a RAS-JNK/p38-dependent pathway in leukemia (37). This report is in accordance with our finding that phospho-JNK/p38 was increased along with ferroptosis in ICH. Furthermore, 20-HETE inhibition decreased the level of phospho-p38 MAPK (Figure 6D), in line with results from vascular smooth muscle cells, in which 20-HETE activated p38 MAPK (52). Inhibition of the JNK pathway by HET0016 (Figure 6D) can also be seen in ischemic stroke (24). Because 20-HETE synthesis inhibition can suppress ICH-induced enhancement of MAPK signaling and biochemical features of ferroptosis, it is of interest to discern whether MAPK pathway suppression regulated by 20-HETE inhibition is an upstream signal to reduce ferroptosis after ICH. In addition, it was reported that active MAPK signaling can sensitize tumors to ferroptosis upon cystine depletion partly because of reduced GPX4 expression (53). The relationship between MAPK pathway activation and GPX4 level in ferroptosis after ICH remains unknown and requires further investigation.

One limitation of this study is that although we carried out *ex vivo* experiments using OHSCs from both sexes, we restricted the *in vivo* experiments to male mice knowing that in some tissue, androgens can drive the expression of CYP 4A synthetic enzymes (54). If the synthesis of 20-HETE is not upregulated to the same extent in females after ICH, the role of 20-HETE–induced ferroptosis may be diminished. Another limitation is that we did not distinguish which cell type is the main source of 20-HETE after ICH. Neurons, astrocytes, microglia, and vascular smooth muscle are all capable of synthesizing 20-HETE, and although cell death in OHSCs occurs primarily in neurons, we cannot exclude the role of intercellular signaling in producing ferroptotic cell death. A third limitation is that, although we demonstrated positive effects of HET0016 on indirect biomarkers of ferroptosis (upregulation of GPX4 and decreased mobile iron, MDA, HNE, and MAPK signaling), we did not provide more direct evidence of effects of HET0016 on neuronal organelle morphology and other biochemical alterations associated with ferroptosis. Nevertheless, our *in vitro* data showing that ferrostatin blocks cell death induced by the 20-HETE stable mimetic supports the concept that 20-HETE is capable of inducing ferroptosis. Lastly, because our previous work showed that HET0016 reduced the number of Iba1- and CD68-positive cells in the peri-lesion tissue (28), we cannot exclude effects of HET0016 on neuroinflammation that could possibly stimulate ferroptosis in neurons in the context of ICH.

## Conclusion

These findings indicate that 20-HETE participates in ICH-induced acute brain injury and neurotoxicity, at least in part, by facilitating ferroptosis, which lays an additional theoretical foundation for the prevention of ICH-induced neurotoxicity. 20-HETE–induced ferroptosis after ICH may be a novel target for therapeutic intervention in ICH.

## Data Availability Statement

The raw data supporting the conclusions of this article will be made available by the authors, without undue reservation.

## Ethics Statement

The animal study was reviewed and approved by Johns Hopkins University Animal Care and Use Committee.

## Author Contributions

RH, JW, XH, Z-JY, and RK designed the study. RH performed the experiments and acquired data and wrote the draft of the manuscript. HR helped acquire data. JF and SM designed and synthesized the 20-HETE analogs and provided advice on the experimental design. RH and RK analyzed the data. RK edited the manuscript. All authors contributed to the article and approved the submitted version.

## Funding

This research was supported by the National Institutes of Health (NS102899, NS102583, and NS105894 to RK), an American Heart Association Postdoctoral Fellowship Award 18POST33970007 to JW, and the Robert A. Welch Foundation (I-001) to JF.

## Conflict of Interest

The authors declare that the research was conducted in the absence of any commercial or financial relationships that could be construed as a potential conflict of interest.

## Publisher's Note

All claims expressed in this article are solely those of the authors and do not necessarily represent those of their affiliated organizations, or those of the publisher, the editors and the reviewers. Any product that may be evaluated in this article, or claim that may be made by its manufacturer, is not guaranteed or endorsed by the publisher.

## Abbreviations

ICH: intracerebral hemorrhage
Hb: hemoglobin
ROS: reactive oxygen species
GPX: glutathione peroxidase
GSH: glutathione
MAPK: mitogen-activated protein kinase
20-HETE, 20-hydroxy-5, 8, 11: 14-eicosatetraenoic acid
CYP: cytochrome P450
HET0016: N-hydroxy-N′-(4-n-butyl-2-methylphenyl)-formamidine
MDA: malondialdehyde
4-HNE: 4-hydroxy-2-nonenal
OHSCs: organotypic hippocampal slice cultures
PI: propidium iodide
20-5, 14–HEDGE, (20-hydroxyeicosa-5(Z): 14(Z)-dienoyl)glycine
20-6, 15–HEDGE, N-(20-hydroxyeicosa-6(Z): 15(Z)-dienoyl)glycine
CV: cresyl violet
ERK: extracellular signal- regulated kinase
JNK: c-Jun NH2-terminal kinase.
